# Cross-cultural adaptation and validation of the Spanish sensory processing sensitivity questionnaire (S-SPSQ)

**DOI:** 10.3389/fpsyg.2024.1279889

**Published:** 2024-05-02

**Authors:** Pedro J. Salinas-Quintana, Rodolfo Barría-Ramírez, Bianca P. Acevedo, Alejandro Vega-Muñoz, Manuela Pérez-Chacón, Antonio Chacón

**Affiliations:** ^1^Faculty of Medicine and Health Sciences, Universidad Central de Chile, Santiago, Chile; ^2^Department of Mathematics and Computer Science, Universidad de Santiago de Chile, Santiago, Chile; ^3^Department of Psychological and Brain Sciences, University of California, Santa Barbara, Santa Barbara, CA, United States; ^4^Facultad de Ciencias Empresariales, Universidad Arturo Prat, Iquique, Chile; ^5^Spanish Association of Highly Sensitive Professionals and Psychologists, PAS España, Madrid, Spain

**Keywords:** psychology, highly sensitive person, sensory processing sensitivity, aesthetic sensitivity, SPSQ

## Abstract

Sensory Processing Sensitivity (SPS) is a temperament trait rooted in biology, and is distinguished by heightened awareness, emotional responsiveness, and sensitivity to environmental stimuli. In this study, we aimed to enable the assessment of SPS within Spanish-speaking populations. To achieve this, we translated, adapted, and validated the Sensory Processing Sensitivity Questionnaire (SPSQ), which offers a comprehensive evaluation of SPS, encompassing both positive and negative aspects of the trait. Participants were 1,004 (844 females, mean age 37) mainly from Chile (964), and 40 were from other Spanish-speaking regions. Confirmatory factor analysis, utilizing the diagonally weighted least squares method, was applied to validate the internal structure of the Spanish version of the SPSQ (S-SPSQ). Fit indices such as GFI, CFI, TLI, RMSEA, and SRMR were scrutinized. Reliability assessment utilized Cronbach’s alpha and McDonald’s Omega. Three models were examined: Model I (six factors) displayed robustness, Model II (six factors plus a general factor) did not show substantive improvement, and Model III (Higher Order and Bifactor) excelled in fit while balancing complexity and representation, thus validating the findings of the original SPSQ and indicating similar reliability coefficients. The study offers a balanced perspective on SPS and contributes to cross-cultural validation of an SPS instrument which may facilitate research and guide personalized therapeutic interventions, thus enhancing outcomes for highly sensitive persons.

## 1 Introduction

Sensory Processing Sensitivity (SPS) is a biologically-based trait that is associated with enhanced awareness of and responsiveness to stimuli in the environment (Acevedo et al., 2018). Research suggests that behaviourally, SPS is characterized by greater caution and inhibition in response to novel stimuli which appears in approximately 20% of humans (Lionetti et al., 2019) and in more than 100+ animal species (Wolf et al., 2008). From an ethological perspective, SPS may be a factor associated with greater adaptation, given that a greater sensitivity and responsiveness to the environment and social stimuli may provide evolutionary advantages (Gearhart and Bodie, 2012; Acevedo et al., 2014).

In 1991, psychologist Elaine Aron introduced the theory of SPS, coining the term the “Highly Sensitive Person” (HSP) to describe a unique group of individuals with high SPS (Aron, 1997). HSPs are characterized by several key features including: (a) heightened awareness and responsiveness to environmental stimuli, (b) deep information processing, (c) heightened emotional reactivity to certain stimuli, and (d) an awareness of subtle details in the environment (Jagiellowicz et al., 2011; Aron and Aron, 2016). The 27-item HSP Scale developed by Aron and Aron (1997) is widely used in studies examining high sensitivity, and it has been shown to be unidimensional, and have satisfactory reliability and validity (Aron et al., 2012). While the HSP Scale is widely used in SPS research it tends to show high associations with negative affectivity (sometimes called neuroticism). It has been suggested that this may be due to the over-sampling of negative items with the HSP Scale (Acevedo, 2024).

Numerous studies have shown some of the positive outcomes associated with SPS, such as openness to experience (Aron, 1997), aesthetic sensitivity (Bröhl et al., 2020; Bröhl and Schury, 2020; De Gucht et al., 2022), resilience (Golonka and Gulla, 2021), and positive responsivity to interventions (for review see Greven et al., 2019). Studies have also found that high sensory sensitivity is associated with negative outcomes such as negative mood states (Amemiya et al., 2020), stress (Ermer and Dunn, 1998; Benham, 2006; Greven et al., 2019; Bas et al., 2021), burnout syndrome (Golonka and Gulla, 2021), propensity to experience nightmares (Carr et al., 2021), introversion and inhibition (Aron et al., 2010, 2012; Listou Grimen and Diseth, 2016), anxiety (Bordarie et al., 2022), shyness and depression (Aron et al., 2012; Karaca Dinç et al., 2021), seasonal depression (Hjordt and Stenbæk, 2019), obsessive-compulsive disorder (Holm et al., 2019), and autism symptoms (Liss et al., 2008). As such, a considerable body of work suggests that there are diverse outcomes associated with SPS.

Also, several psychometric studies have contributed to a deeper understanding of SPS, revealing its complexity and robustness (Carlson and Doyle, 2002). For example, the research by Smolewska et al. (2006) was the first to identify three underlying SPS dimensions: ease of excitation (EOE), low sensory threshold (LST), and aesthetic sensitivity (AES). AES is characterized by a strong interest in art, an intense emotional appreciation of beauty, and a notable creative potential (Smolewska et al., 2006; Bröhl and Schury, 2020; Bröhl et al., 2020; Khosravani et al., 2021; De Gucht et al., 2022). EOE is the tendency to be responsive to both negative and positive stimuli, and has been found to be positively correlated with anxiety and depression (Liss et al., 2005; Bakker and Moulding, 2012). Also, both EOE and LST (which is the tendency to become aroused by low levels of a stimulus) were found to be significantly associated with avoidant personality disorder (Meyer and Carver, 2000) and social phobia (Neal et al., 2002). These studies found that the three dimensions of SPS were more stable than a unifactorial solution of SPS.

Other studies, such as those by Evans D. E. and Rothbart (2007), Evans D.E. and Rothbart (2009), and Evans and Rothbart (2008), have also found a non-unifactorial structure for SPS. In a study with 297 university students, Evans D. E. and Rothbart (2007) and Evans D.E. and Rothbart (2009) conducted a confirmatory factor analysis using the maximum likelihood method. The study found a two-factor model for SPS comprising sensory sensitivity and sensory discomfort. Their findings indicated that the two dimensions were not correlated and suggested that the HSP Scale primarily consisted of items reflecting distinct constructs of negative affect and orienting sensitivity. Similar results were found by Ershova and Berezina (2018), Lionetti et al. (2018) and Lionetti et al. (2019) measuring SPS with the HSP Scale, and finding a bi-factor structure.

Studies of SPS conducted in diverse cultural contexts also provide support for a multidimensional factor structure of the HSP Scale (Aron, 1997; Smolewska et al., 2006; Evans D. E. and Rothbart, 2007; Sadoughi et al., 2007; Evers et al., 2008; Evans D.E. and Rothbart, 2009; Listou Grimen and Diseth, 2016; Ershova et al., 2018; Lionetti et al., 2018; 2019; Khosravani et al., 2021). For example, Sadoughi et al. (2007) found a three-factor structure for the HSP scale in a sample of Iranian university students. Khosravani et al. (2021), using the Persian version of the HSP Scale (consisting of 25 items), also found a three-factor structure for the HSP Scale, consisting of AES, sensory overstimulation, and LST—similar to that found by Smolewska et al. (2006). Evers et al. (2008) and Listou Grimen and Diseth (2016) found that versions of the HSP Scale with 13 and 18 items showed three factors and demonstrated adequate reliability. Chacón et al. (2021) conducted a study with 8,358 participants, adapting and validating the HSP Scale in Spanish, while also examining its psychometric properties. Using factor analyses the study confirmed a Spanish version of the 27-item HSP Scale and found five dimensions: sensitivity to overstimulation (SOS), aesthetic sensitivity (AES), low sensory threshold (LST), fine psychophysiological discrimination (FPD), and harm avoidance (HA). The five-factor structure demonstrated invariance across gender, and the reliability indices indicated good internal consistency. Konrad and Herzberg (2019) also found a multidimensional structure of the HSP Scale among a German general population sample. Montoya-Pérez et al. (2019) who also made an adaptation to Spanish, but specifically for the Mexican population, and also found a multidimensional structure of the HSP Scale. Also, a study by Chacón et al. (2021) among a Spanish population also found support for a multidimensional structure of the HSP Scale. Bordarie et al. (2022) validated and investigated the psychometric properties of a French version of the HSP Scale and also found a multidimensional structure. The short, Polish version of the HSP Scale (Baryła-Matejczuk et al., 2023) also found evidence of a multidimensional structure. Lionetti et al. (2024) validated the psychometric properties of a short version of the HSP Scale (HSP-12) for the United Kingdom and Italy with multiple samples of adults (*N* = 4,459). De Gucht et al. (2023) among a Dutch sample found that a bifactor model, consisting of one overall factor and three separate factors, provided the best fit to the data for each sample. The three distinct factors, which encompassed various dimensions of SPS were Ease of Arousal, Sensory and Aesthetic Sensitivity, and Low Sensory Threshold. In sum, these studies suggest the presence of a multidimensional structure of SPS, supporting varying phenotypes of one overall SPS trait (e.g., Aron and Aron, 1997; Bolders et al., 2017).

Additionally, and irrespective of the scale used, there are numerous psychometric studies of SPS that have been conducted globally in recent years to establish the construct validity of SPS. The adaptations have varied in the number of items used, typically ranging from 10 (e.g., Limura et al., 2022) to 60 items (e.g., De Gucht et al., 2022). The validation samples have included diverse populations with sample sizes ranging from approximately 150 individuals to 10,800. The HSP Scale has also been adapted and validated for youth (e.g., ages 6–19 years). Also, research by Costa-López et al. (2022) and Flores et al. (2023), among others, focused on Spanish samples.

While the HSP scale is widely used, it has limitations, including its bias toward negative aspects of the SPS trait. Thus, in the present study, we utilized the Sensory Processing Sensitivity Questionnaire (SPSQ)—which has six dimensions that measure both the positive and negative aspects of high sensitivity (De Gucht et al., 2022)—to capture the underlying structure of SPS among a Spanish-speaking sample. To achieve this objective, we translated and adapted the SPSQ for an adult Spanish-speaking population in Chile and other Spanish-speaking countries. The second aim of this study was to examine the psychometric properties of the adapted SPSQ, including its factor structure and reliability, within a Spanish speaking sample. By pursuing these objectives, we aimed to confirm the validity and reliability of the Spanish SPSQ (S-SPSQ) in a Spanish-speaking adult population. To meet these objectives, a team of experts first translated and adapted the SPSQ following guidelines by Hambleton and Patsula (1998) and Hambleton and Li (2005). Then, we conducted a pilot study of the S-SPSQ with an online sample to obtain feedback. We then modified the S-SPSQ according to the feedback and investigated the factor structure of the final S-SPSQ testing three different models: Model I, with six specific factors; Model II, a bifactor model incorporating a general factor; and Model III, combining a higher-order model and a bifactorial model.

## 2 Materials and methods

### 2.1 Procedure

The initial phase of the research project involved obtaining approval from both the university and the institutional ethics committees. First, we translated and adapted the SPSQ (De Gucht et al., 2022) to Spanish following the guidelines of Hambleton and Patsula (1998) and Hambleton and Li (2005). The adaptation and translation into Spanish were conducted by a team of four bilingual experts who participated in all stages of the process.

Subsequently, a pilot test of the S-SPSQ was conducted with 88 university students who volunteered to participate in the study through the university’s internal communication channels. The participants were provided with a Google form that included an informed consent and the S-SPSQ. They were given the opportunity to provide feedback on any ambiguous items.

The main study was carried out between April and December 2022, following a procedure like the pilot phase. Participants provided informed consent and an online application was utilized for survey completion with an expected completion time of 10–15 min.

### 2.2 Participants

The participants were 1,004 individuals (844 females and 160 males) from Chile and other Spanish-speaking countries, with a mean age of 36.9 years (*SD* = 12.41, range 18–85 years). Nine-hundred and sixty-four were of Chilean nationality (with 712 residing in the metropolitan region and 252 in other regions of Chile). Forty participants were residing abroad, specifically in Argentina, Colombia, Mexico, Spain, and Uruguay. Regarding education, the sample included 119 (11.85%) university technicians, 507 (50.50%) university students, and 318 (37.45%) individuals with postgraduate education. A small proportion, 0.20%, did not provide information about their educational background.

### 2.3 Linguistic validation

The translation and adaptation process of the SPSQ (De Gucht et al., 2022) followed the guidelines of Hambleton and Patsula (1998) and Hambleton and Li (2005). The initial translation of the 60 items into Spanish was conducted by two English language professionals, a translator, and an English language teacher, both native Spanish speakers. Neither of the translators were affiliated with the research team and were impartial to the study’s outcomes. Any semantic discrepancies between the translators were resolved in collaboration with the research team. Subsequently, a reverse translation was performed with the assistance of two other bilingual translators (English–Spanish) who are native English speakers. Based on their feedback, two of the 60 items were reformulated. The first team of translators then conducted a third translation, incorporating the reformulations of the observed item. The resulting version was evaluated by a focus group consisting of 12 volunteers over 18 years of age. The focus group assessed readability and content, providing positive feedback without identifying any readability or comprehension issues in any of the items. This process resulted in the first 60-item Spanish version of the SPSQ (S-SPSQ).

### 2.4 Instrument

The SPSQ by De Gucht et al. (2022) is a self-report instrument designed to measure the degree of high sensitivity in adults. It consists of 60 items with Likert-type responses ranging from 1 (not at all) to 7 (extremely). In the study conducted by De Gucht et al. (2022), a higher order bifactor model was confirmed, consisting of two higher order factors that represent positive and negative dimensions of SPS. In addition, 6 dimensions were identified as follows: (1) Sensory Sensitivity to Subtle Internal and External Stimuli (SIES)(+); (2) Emotional and Physiological Reactivity (EPR)(−); (3) Sensory Discomfort (SD)(−); (4) Sensory Comfort (SC)(+); (5) Socio-Affective Sensitivity (SAS)(+); and (6) Aesthetic Sensitivity (AS)(+).

The questionnaire designed by De Gucht et al. (2022) had factor loadings with significant magnitudes greater than 0.4, as shown by confirmatory factor analysis. Cronbach’s αs were adequate for each of the factors between (*α* = 0.75–0.90), except for Sensory Comfort (*α* = 0.62). The positive dimension of the SPSQ (SIES, SC, SAS, AS) and the negative dimension of the (EPR and SD) presented good reliability (*α* = 0.90–0.93), and the fit indices obtained for this instrument (CFI = 0.985, SRMR = 0.057, and RMSEA = 0.067) indicated a good model fit. The invariance of the SPSQ between sociodemographic groups was verified. When considering sociodemographic groups, no significant change in model fit was observed when restricting loadings and then intercepts. In each case, the changes in CFI, SRMR and RMSEA did not exceed the criteria proposed by Chen (2007).

### 2.5 Data analysis

The main aim of our study was to confirm the latent structure of the S-SPSQ through three separate instances of confirmatory factor analysis (CFA). In the first instance, a first-order CFA model was fit, consisting of the factors AS = Aesthetic Sensitivity, SAS = Social-Affective Sensitivity, SIES = Sensory Sensitivity to Subtle Internal and External Stimuli, SC = Sensory Comfort/Pleasure, EPR = Emotional and Physiological Reactivity, and SD = Sensory Discomfort. In this structure, the factors were allowed to correlate, as their original configuration is based on an oblique rotation. Second, a bifactor model was fit to investigate whether the latent structure of the SPSQ suggested a general SPS factor, along with the specific factors identified in the previous objective. In this case, the factors were orthogonal. In the third instance, a higher-order bifactor model was fit, consisting of the items of the bifactor model plus two higher-order positive (POS) and negative (NEG) dimensions of SPS. In sum, in our exploration of the translated and adapted version of SPSQ for Spanish-speaking persons, three models were contrasted: Model I, with six specific factors; Model II, a bifactor model incorporating a general factor; and Model III, combining a higher-order model and a bifactorial model. The results indicated that Model III outperformed the others in fit, achieving an optimal balance between complexity and detailed representation of sensory sensitivity.

It is important to note that the Likert scaling employed in the SPSQ assumes a continuous latent structure, even though the observed data are measured at an ordinal level (Muthén and Kaplan, 1985). Consequently, robust methods based on weighted least squares (WLS) were implemented (Mair and Wilcox, 2020). The parameters of the Confirmatory Factor Analysis (CFA) model were estimated using the diagonally weighted least squares (DWLS) method, with the first indicator of each factor set to 1, as recommended by Muthén et al. (1997) and Sun (2005). To evaluate the fit between the proposed model and empirical data, various indices have been employed, including the Goodness-of-Fit Index (GFI), Root Mean Square Error of Approximation (RMSEA), Standardized Root Mean Square Residual (SRMR), Comparative Fit Index (CFI), and Tucker-Lewis Index (TLI), as advocated in the literature (Tucker and Lewis, 1973; Schreiber et al., 2006) (refer to Table 1 for details).

**Table 1:** Cutoff criterial for several fit indexes.

Indexes	Shorthand	General rule for acceptable fit
Comparative fit		Comparison to a baseline or other model
Comparative fit index	CFI	≥0.95 for acceptance
Tucker-Lewis index	TLI	≥0.95 for acceptance
Other		
Goodness of fit index	GFI	≥0.95 for acceptance
Root mean square residual Standardized	SRMR	≤0.08 for acceptance
Root mean square error of approximation	RMSEA	<0.05 or <0.08 for acceptance

From Schermelleh-Engel et al. (2003) and Schreiber et al. (2006).

The alpha index serves as an estimate of the total score reliability, assuming a single construct for all questionnaire items. Nevertheless, it relies on certain assumptions: (1) the factor model is well-specified (indicating a unidimensional questionnaire), (2) factor loadings of the items are equal (essential tau-equivalence), and (3) errors are independent across items. Deviations from essential tau-equivalence can introduce significant bias to alpha reliability estimates, particularly when items exhibit both positive and negative loadings in the factor. Moreover, the omega index assesses the reliability of the model factor(s) by adjusting the essential tau-equivalence approach to a congeneric approach with fewer restrictive assumptions. Omega is robust to variations in factor loadings within a factor and remains unaffected by biases in item distributions. This unique characteristic enables omegas to effectively address the limitations inherent in Cronbach’s alpha, as explained in Flora (2020). Therefore, our analytical strategy adopts McDonald’s omega coefficient, instead of the coefficient alpha used by De Gucht et al. (2022) in the original SPSQ.

The reliability assessment employed the McDonald’s Omega coefficient (ω), which offers more precise estimates compared to Cronbach’s alpha, as it is based on factor loadings derived from the matrix of polychoric correlations (Revelle and Zinbarg, 2009). This coefficient was calculated for all factors within each model. To evaluate the relationships between items and factors, the sign, magnitude, confidence interval, and statistical significance of the loadings were inspected. Confidence intervals were obtained using a bootstrap adjusted bias-corrected method, with a total of 500 bootstrap samples. The statistical data were performed using Jamovi version 2.3 and/or R version 4.1.2 (Jorgensen et al., 2019).

### 2.6 Ethics considerations

The study adhered to ethical guidelines as outlined in the Declaration of Helsinki. The research protocol received approval from the Universidad Central de Chile’s Faculty of Health, Research Institute, and Ethics Committee (Project identification code 120/2022). To ensure confidentiality, consent forms and instruments were collected separately.

Ethical considerations were based on the principles set forth by the World Medical Association (2013) and the Code of Ethics of the Colegio de Psicólogos de Chile (1987), and the recommended ethical guidelines for research involving human participants. The research group declared no conflicts of interest. Participants were fully informed about the study objectives and assured of anonymity and data protection. Additionally, the participants were provided the opportunity to provide feedback upon completion of the study. A contact platform, including a Google Forms link and email contact with the project director, was made available for this purpose.

## 3 Results

The proposed model comprised a first-order latent structure consisting of six individual factors. In the initial analysis, we examined the standardized factor loadings $\lambda_{ij}$ as an integral component of the theoretical model. The loadings indicate the extent to which the latent factor *i* is associated with item *j*, and the squared value $\lambda_{ij}^{2}$ represents the variance of item *j* explained by latent factor *i*. Adequate factor loadings are considered to be those with confidence intervals $CI\left(\lambda_{ij};95\%\right)$ that do not include zero, indicating positive lower and upper limits (Aldas and Uriel, 2017).

Model I was formed by a first-order latent structure grouped into six individual factors. Factor loadings (standardized) were found with an average magnitude equal to 0.688 and a range that varied between 0.395 and 0.947, except for item 6 (“I cannot enjoy the little things in life”) which obtained a loading of λ = −0.169, accounting for the smallest absolute loading, and is considered an atypical result. To avoid biases in the statistical indices of goodness-of-fit and reliability, this item was omitted from the analysis.

Model II comprised a first-order latent structure with six specific factors and a general factor. It assumed the six factors identified by De Gucht et al. (2022) and the removal of item 6 from the Comfort/Sensory Pleasure (SC) factor and from the general factor (g). The standardized loadings were all positive, with an average magnitude of 0.479, and a range varying from 0.082 to 0.798.

Model III consisted of a higher-order bifactor latent structure comprising: six specific factors, one general factor, and two secondary factors (a positive and a negative one). Notably, item 6 was not included in the factor arrangement for Sensory Comfort/Pleasure (SC), the general factor (g), or the second-order positive dimension.

The standardized loadings were predominantly positive and significant. However, exceptions were noted with items 26 and 30, both of which belong to the g factor, exhibiting non-significant standardized loadings with 95% confidence intervals encompassing zero. On average, the magnitude was 0.493, ranging from −0.062 to 0.888.

### 3.1 Descriptive and inferential analysis: scale performance

Below, we present the descriptive statistics for the standardized factor loadings of each factor in Models I, II and III including the mean, minimum, maximum, and range of variation.

#### 3.1.1 Model I

- Sensory Discomfort (SD): the mean factor loading is 0.805, with a minimum value of 0.556 and a maximum of 0.947, resulting in a range of variation of 0.391.
- Aesthetic Sensitivity (AS): the mean factor loading is 0.717, with a minimum value of 0.626 and a maximum of 0.779, yielding a range of variation of 0.153.
- Social Affective Sensitivity (SAS): The mean factor loading is 0.686, with a minimum value of 0.510 and a maximum of 0.848, resulting in a range of variation of 0.338.
- Emotional and Physiological Reactivity (EPR): The mean factor loading is 0.667, with a minimum value of 0.510 and a maximum of 0.835, leading to a range of variation of 0.157.
- Sensory Sensitivity to External Subtle Stimuli (SIES): The mean factor loading is 0.625, with a minimum of 0.532 and a maximum of 0.691, resulting in a range of variation of 0.159.
- Sensory Comfort/Pleasure (SC): This factor exhibits the lowest loadings, with a mean of 0.577, a minimum of 0.395, and a maximum of 0.872, reflecting the highest range of variation at 0.477.

It is noteworthy that the estimated factor loadings are significantly different from zero (*p* < 0.05), and the bootstrap CI method ($\lambda_{ij}$; 95%) has estimated the lower and upper limits of the confidential interval with positive values. Consequently, these findings underscore the substantial contribution of the items to the variability of the constituent factors within the model (Table 2).

**Table 2:** Estimations of factor loadings for model I.

				Std. Est		
				CI 95%		
Latent	Indicator	Estimate	Std. Est.	Lower	Upper	*p*-value
AS	i28	1.000	0.626	0.603	0.648	
	i12	1.210	0.757	0.736	0.778	< 0.001
	i51	1.044	0.654	0.633	0.674	< 0.001
	i22	1.245	0.779	0.758	0.800	< 0.001
	i54	1.225	0.767	0.747	0.787	< 0.001
SAS	i50	1.000	0.511	0.490	0.532	
	i49	1.434	0.733	0.713	0.752	< 0.001
	i56	1.114	0.569	0.549	0.589	< 0.001
	i4	1.533	0.783	0.764	0.803	< 0.001
	i21	1.372	0.701	0.683	0.719	< 0.001
	i14	1.278	0.653	0.633	0.673	< 0.001
	i5	1.349	0.690	0.669	0.710	< 0.001
	i36	1.658	0.848	0.830	0.865	< 0.001
SIES	i35	1.000	0.684	0.662	0.705	
	i23	0.886	0.606	0.584	0.628	< 0.001
	i43	0.908	0.621	0.600	0.642	< 0.001
	i44	0.779	0.532	0.511	0.553	< 0.001
	i24	1.010	0.691	0.669	0.713	< 0.001
	i37	0.902	0.617	0.595	0.638	< 0.001
SD	i33	1.000	0.765	0.748	0.782	
	i7	1.106	0.846	0.833	0.859	< 0.001
	i9	0.726	0.555	0.536	0.574	< 0.001
	i11	1.032	0.790	0.776	0.804	< 0.001
	i25	1.238	0.947	0.935	0.960	< 0.001
	i48	1.093	0.836	0.820	0.852	< 0.001
	i10	1.040	0.796	0.779	0.813	< 0.001
	i42	1.186	0.908	0.895	0.920	< 0.001
EPR	i40	1.000	0.650	0.631	0.669	
	i3	1.232	0.801	0.782	0.819	< 0.001
	i46	0.957	0.622	0.603	0.640	< 0.001
	i19	1.167	0.758	0.739	0.777	< 0.001
	i60	0.867	0.564	0.544	0.583	< 0.001
	i47	1.088	0.707	0.687	0.728	< 0.001
	i34	1.087	0.707	0.687	0.726	< 0.001
	i59	1.285	0.836	0.818	0.853	< 0.001
	i55	0.784	0.510	0.488	0.531	< 0.001
	i2	0.949	0.617	0.599	0.635	< 0.001
	i18	0.868	0.565	0.546	0.584	< 0.001
SC	i30	1.000	0.492	0.461	0.522	
	i15	1.259	0.619	0.586	0.652	< 0.001
	i26	0.797	0.392	0.362	0.422	< 0.001
	i53	1.779	0.875	0.834	0.916	< 0.001

Estimated factor loadings. Model I consist of six factors: AS, aesthetic sensitivity; SAS, social-affective sensitivity; SIES, sensory sensitivity to subtle internal and external stimuli; SC, sensory comfort/pleasure; EPR, emotional and physiological reactivity; SD, sensory discomfort. In the configuration of the factors in Model I, correlations were allowed among them since their original configuration is based on an oblique rotation.

#### 3.1.2 Model II

- Sensory Discomfort (SD): the mean factor loading is 0.622, with a minimum value of 0.486 and a maximum of 0.798, resulting in a range of variation of 0.312.
- Aesthetic Sensitivity (AS): the mean factor loading is 0.367, with a minimum value of 0.082 and a maximum of 0.613, leading to a range of variation of 0.531.
- Social Affective Sensitivity (SAS): the mean factor loading is 0.441, with a minimum value of 0.246 and a maximum of 0.601, resulting in a range of variation of 0.355.
- Emotional and Physiological Reactivity (EPR): the mean factor loading is 0.528, with a minimum value of 0.212 and a maximum of 0.713, yielding a range of variation of 0.501.
- Sensory Sensitivity to External Subtle Stimuli (SIES): the mean factor loading is 0.295, with a minimum of 0.090 and a maximum of 0.449, leading to a range of variation of 0.359.
- Sensory Comfort/Pleasure (SC): this factor exhibits a mean loading of 0.522, a minimum of 0.373, and a maximum of 0.593, resulting in a range of variation of 0.220.
- General Factor (g): the mean loading is 0.481, with a minimum of 0.154 and a maximum of 0.664, resulting in a range of variation of 0.510.

The estimated factor loadings are significantly different from zero (*p* < 0.05), and the bootstrap CI method ($\lambda_{ij}$; 95%) has calculated the lower and upper limits of the confidential interval with positive values. The items also significantly contribute to the variability of the constituent factors of the model. However, noteworthy is the observed substantial decrease in the performance of the S-SPSQ concerning Model I (see Table 3).

**Table 3:** Estimations of factor loadings for model II.

				Std. Est		
				CI 95%		
Latent	Indicator	Estimate	Std. Est.	Lower	Upper	*p*-value
AS	i28	1.000	0.082	0.012	0.153	
	i12	2.757	0.227	0.169	0.286	0.027
	i51	4.350	0.359	0.297	0.420	0.025
	i22	7.443	0.613	0.520	0.707	0.024
	i54	6.735	0.555	0.470	0.640	0.024
SAS	i50	1.000	0.298	0.247	0.349	
	i49	1.315	0.392	0.346	0.438	< 0.001
	i56	1.552	0.462	0.413	0.512	< 0.001
	i4	1.698	0.506	0.460	0.552	< 0.001
	i21	1.671	0.498	0.453	0.543	< 0.001
	i14	1.770	0.527	0.478	0.576	< 0.001
	i5	0.825	0.246	0.198	0.294	< 0.001
	i36	2.017	0.601	0.556	0.646	< 0.001
SIES	i35	1.000	0.347	0.265	0.428	
	i23	1.018	0.353	0.267	0.439	< 0.001
	i43	1.161	0.403	0.316	0.490	< 0.001
	i44	1.295	0.449	0.353	0.546	< 0.001
	i24	0.375	0.130	0.053	0.207	0.002
	i37	0.260	0.090	0.012	0.169	0.029
SD	i33	1.000	0.523	0.492	0.554	
	i7	1.305	0.683	0.659	0.706	< 0.001
	i9	0.930	0.486	0.452	0.521	< 0.001
	i11	1.229	0.643	0.618	0.668	< 0.001
	i25	1.526	0.798	0.775	0.822	< 0.001
	i48	0.973	0.509	0.481	0.537	< 0.001
	i10	1.087	0.568	0.537	0.600	< 0.001
	i42	1.463	0.765	0.741	0.788	< 0.001
EPR	i40	1.000	0.713	0.681	0.744	
	i3	0.990	0.705	0.676	0.735	< 0.001
	i46	0.941	0.670	0.640	0.701	< 0.001
	i19	0.610	0.435	0.404	0.465	< 0.001
	i60	0.806	0.575	0.543	0.606	< 0.001
	i47	0.313	0.223	0.189	0.257	< 0.001
	i34	0.595	0.424	0.393	0.455	< 0.001
	i59	0.821	0.585	0.557	0.613	< 0.001
	i55	0.297	0.212	0.177	0.247	< 0.001
	i2	0.988	0.704	0.673	0.735	< 0.001
	i18	0.793	0.565	0.534	0.596	< 0.001
SC	i30	1.000	0.562	0.488	0.636	
	i15	1.056	0.593	0.517	0.670	< 0.001
	i26	0.998	0.561	0.486	0.637	< 0.001
	i53	0.663	0.373	0.306	0.439	< 0.001
g	i12	1.000	0.664	0.645	0.683	
	i28	0.831	0.552	0.531	0.573	< 0.001
	i51	0.819	0.544	0.524	0.564	< 0.001
	i22	0.939	0.624	0.602	0.645	< 0.001
	i54	0.925	0.614	0.594	0.635	< 0.001
	i50	0.583	0.387	0.365	0.409	< 0.001
	i49	0.884	0.587	0.566	0.607	< 0.001
	i56	0.598	0.397	0.376	0.418	< 0.001
	i4	0.898	0.596	0.575	0.618	< 0.001
	i21	0.781	0.519	0.498	0.539	< 0.001
	i14	0.686	0.456	0.434	0.477	< 0.001
	i5	0.901	0.598	0.576	0.621	< 0.001
	i36	0.952	0.632	0.613	0.652	< 0.001
	i35	0.901	0.598	0.579	0.618	< 0.001
	i23	0.789	0.524	0.502	0.545	< 0.001
	i43	0.806	0.535	0.515	0.555	< 0.001
	i44	0.677	0.450	0.430	0.470	< 0.001
	i24	0.942	0.625	0.606	0.645	< 0.001
	i37	0.854	0.567	0.547	0.587	< 0.001
	i33	0.795	0.528	0.506	0.549	< 0.001
	i7	0.763	0.507	0.486	0.528	< 0.001
	i9	0.479	0.318	0.296	0.340	< 0.001
	i11	0.718	0.477	0.456	0.498	< 0.001
	i25	0.800	0.531	0.510	0.552	< 0.001
	i48	0.935	0.621	0.600	0.642	< 0.001
	i10	0.810	0.538	0.516	0.560	< 0.001
	i42	0.766	0.508	0.487	0.530	< 0.001
	i40	0.401	0.266	0.245	0.288	< 0.001
	i3	0.672	0.446	0.426	0.467	< 0.001
	i46	0.391	0.260	0.239	0.280	< 0.001
	i19	0.829	0.551	0.531	0.571	< 0.001
	i60	0.404	0.269	0.247	0.290	< 0.001
	i47	0.927	0.616	0.596	0.635	< 0.001
	i34	0.789	0.524	0.504	0.544	< 0.001
	i59	0.847	0.563	0.543	0.582	< 0.001
	i55	0.638	0.424	0.403	0.445	< 0.001
	i2	0.346	0.230	0.209	0.251	< 0.001
	i18	0.396	0.263	0.242	0.283	< 0.001
	i30	0.340	0.226	0.205	0.247	< 0.001
	i15	0.535	0.356	0.334	0.377	< 0.001
	i26	0.231	0.154	0.132	0.175	< 0.001
	i53	0.845	0.561	0.541	0.582	< 0.001

Estimated factor loadings. The second model comprises six factors: AS, aesthetic sensitivity; SAS, social-affective sensitivity; SIES, sensory sensitivity to subtle internal and external stimuli; SC, sensory comfort/pleasure; EPR, emotional and physiological reactivity; SD, sensory discomfort, and one general factor (g). The factors are orthogonal.

#### 3.1.3 Model III

- Sensory Discomfort (SD): the factor exhibits loadings with an average magnitude of 0.586, ranging from 0.426 to 0.796 across all its items.
- Aesthetic Sensitivity (AS): this factor displays loadings with an average magnitude of 0.567, ranging from 0.485 to 0.698.
- Social Affective Sensitivity (SAS): the factor reveals loadings with an average magnitude of 0.567, ranging from 0.360 to 0.694.
- Emotional and Physiological Reactivity (EPR): this factor demonstrates loadings with an average magnitude of 0.512, ranging from 0.145 to 0.726.
- Sensory Sensitivity to External Subtle Stimuli (SIES): the factor presents mean loadings of 0.412, ranging from 0.302 to 0.542.
- Sensory Comfort/Pleasure (SC): standardized loadings for this factor have an average magnitude of 0.607, ranging from 0.556 to 0.652.
- General Factor (g): the general factor displays loadings of average magnitude equal to 0.415, ranging from −0.022 to 0.696, with items 26 and 30 showing non-significant loadings (*p* < 0.05).

The bootstrap CI method ($\lambda_{ij}$; 95%) calculation estimated the lower and upper limits of the confidential intervals with positive values, except for items 26 and 30. Therefore, all items, except the 26th and 30th items, significantly contribute to the variability of the model’s constituent factors. Regarding the POS and NEG dimensions, their factor loadings are positive and significant (Table 4).

**Table 4:** Estimations of factor loadings for model III.

				Std. Est. 95% CI		
Latent	Indicator	Estimate	Std. Est.	Lower	Upper	*p*-value
AS	i28	1.000	0.485	0.392	0.579	
	i12	1.162	0.564	0.475	0.654	< 0.001
	i51	1.149	0.558	0.476	0.640	< 0.001
	i22	1.222	0.593	0.503	0.684	< 0.001
	i54	1.438	0.698	0.615	0.781	< 0.001
SAS	i50	1.000	0.518	0.438	0.598	
	i49	1.040	0.539	0.467	0.611	< 0.001
	i56	1.101	0.571	0.503	0.639	< 0.001
	i4	1.178	0.611	0.529	0.693	< 0.001
	i21	1.154	0.598	0.524	0.672	< 0.001
	i14	1.240	0.643	0.572	0.714	< 0.001
	i5	0.694	0.360	0.274	0.446	< 0.001
	i36	1.338	0.694	0.623	0.765	< 0.001
SIES	i35	1.000	0.465	0.361	0.569	
	i23	1.015	0.472	0.369	0.575	< 0.001
	i43	0.649	0.302	0.184	0.419	< 0.001
	i44	0.704	0.327	0.198	0.456	< 0.001
	i24	1.164	0.542	0.443	0.640	< 0.001
	i37	0.775	0.360	0.241	0.479	< 0.001
SD	i33	1.000	0.471	0.361	0.581	
	i7	1.423	0.670	0.589	0.751	< 0.001
	i9	0.904	0.426	0.311	0.540	< 0.001
	i11	1.313	0.618	0.529	0.708	< 0.001
	i25	1.688	0.796	0.701	0.889	< 0.001
	i48	0.967	0.455	0.322	0.589	< 0.001
	i10	1.047	0.493	0.354	0.632	< 0.001
	i42	1.611	0.759	0.670	0.847	< 0.001
EPR	i40	1.000	0.726	0.665	0.787	
	i3	0.960	0.697	0.634	0.760	< 0.001
	i46	0.932	0.676	0.615	0.737	< 0.001
	i19	0.611	0.444	0.345	0.542	< 0.001
	i60	0.778	0.565	0.503	0.627	< 0.001
	i47	0.199	0.145	0.064	0.224	< 0.001
	i34	0.526	0.382	0.308	0.456	< 0.001
	i59	0.787	0.572	0.491	0.652	< 0.001
	i55	0.227	0.165	0.079	0.251	< 0.001
	i2	0.962	0.699	0.633	0.764	< 0.001
	i18	0.771	0.560	0.485	0.634	< 0.001
SC	i30	1.000	0.640	0.557	0.722	
	i15	0.904	0.579	0.502	0.655	< 0.001
	i26	0.868	0.556	0.472	0.639	< 0.001
	i53	1.018	0.652	0.566	0.736	< 0.001
g	i12	1.000	0.480	0.394	0.566	
	i28	0.799	0.384	0.295	0.473	< 0.001
	i51	0.761	0.365	0.278	0.453	< 0.001
	i22	1.004	0.482	0.397	0.567	< 0.001
	i54	0.826	0.397	0.307	0.487	< 0.001
	i50	0.458	0.220	0.128	0.312	< 0.001
	i49	0.972	0.467	0.383	0.550	< 0.001
	i56	0.518	0.249	0.158	0.340	< 0.001
	i4	0.980	0.471	0.384	0.558	< 0.001
	i21	0.795	0.382	0.303	0.460	< 0.001
	i14	0.602	0.289	0.195	0.383	< 0.001
	i5	1.148	0.552	0.474	0.629	< 0.001
	i36	1.009	0.485	0.402	0.567	< 0.001
	i35	1.045	0.502	0.411	0.593	< 0.001
	i23	0.863	0.414	0.323	0.506	< 0.001
	i43	1.074	0.516	0.428	0.604	< 0.001
	i44	0.856	0.411	0.320	0.502	< 0.001
	i24	0.976	0.469	0.380	0.558	< 0.001
	i37	1.011	0.486	0.405	0.567	< 0.001
	i33	1.218	0.585	0.490	0.681	< 0.001
	i7	1.100	0.528	0.450	0.607	< 0.001
	i9	0.764	0.367	0.260	0.474	< 0.001
	i11	1.047	0.503	0.432	0.574	< 0.001
	i25	1.119	0.538	0.464	0.611	< 0.001
	i48	1.425	0.685	0.581	0.788	< 0.001
	i10	1.264	0.607	0.488	0.727	< 0.001
	i42	1.081	0.519	0.443	0.596	< 0.001
	i40	0.514	0.247	0.1598	0.3345	< 0.001
	i3	0.948	0.456	0.3665	0.5454	< 0.001
	i46	0.527	0.253	0.1658	0.3416	< 0.001
	i19	1.213	0.583	0.4928	0.6735	< 0.001
	i60	0.568	0.273	0.1903	0.3564	< 0.001
	i47	1.447	0.696	0.6280	0.7636	< 0.001
	i34	1.173	0.564	0.4936	0.6350	< 0.001
	i59	1.220	0.586	0.5110	0.6625	< 0.001
	i55	0.980	0.471	0.4049	0.5381	< 0.001
	i2	0.475	0.228	0.1275	0.3300	< 0.001
	i18	0.583	0.280	0.1851	0.3756	< 0.001
	i30	−0.047	−0.022	−0.1108	0.0652	0.616
	i15	0.334	0.160	0.0656	0.2562	0.001
	i26	−0.129	−0.062	−0.1566	0.0318	0.212
	i53	0.705	0.339	0.2432	0.4351	< 0.001
POS	AS	1.000	0.887	0.8238	0.9517	
	SAS	0.874	0.727	0.6596	0.7948	< 0.001
	SIES	0.802	0.743	0.6584	0.8293	< 0.001
	SC	1.220	0.822	0.7325	0.9118	< 0.001
NEG	EPR	1.000	0.760	0.6894	0.8305	
	SD	0.321	0.376	0.2487	0.5034	< 0.001

Estimated factor loadings. The third model includes six factors: AS, aesthetic sensitivity; SAS, social-affective sensitivity; SIES, sensory sensitivity to subtle internal and external stimuli; SC, sensory comfort/pleasure; EPR, emotional and physiological reactivity; SD, sensory discomfort, one general factor (g), and two higher-order factors; NEG, negative dimension of S-SPSQ; POS, positive dimension of S-SPSQ. The factors are orthogonal.

### 3.2 Confirmatory factorial analysis

Theoretical models can be conceptualized as a set of hypotheses constrained to a specific domain of phenomena, primarily designed for explanatory purposes. Typically, these hypotheses are derived from fully or partially developed theories or empirical generalizations, forming a structured framework of relationships that can be tested and compared against empirical facts to validate the theoretical model (Hu and Bentler, 1999). Covariances serve as statistical metrics that summarize the relationships between two or more variables comprising the phenomenon. Calculated from a dataset, covariances help reveal the extent to which variables (or more) co-vary, indicating the strength and direction of their relationship. Additionally, they provide insight into how the theory predicts these relationships (Brown, 2015). Model validation occurs when it is confirmed that the observed relationships between variables align with theoretical expectations. The null hypothesis posits that the theoretically implied covariance matrix is equal to the observed covariance matrix, signifying a perfect fit. To assess the disparity between expected and observed values, an adjustment function is employed. This function gauges the extent to which the matrix of theoretically implied covariances aligns with the matrix of observed covariances. Small discrepancies between the matrices may not provide sufficient evidence to challenge the fit between the proposed theoretical model and observed relationships (Aldas and Uriel, 2017).

Model I, using confirmatory factor analysis (CFA), demonstrated a Goodness of Fit Index (GFI) of 0.977. This suggests that 97.7% of the observed variances and covariances are accounted for by the variance–covariance matrix estimated by the model. According to Ruiz et al. (2010) and Schreiber et al. (2006), a GFI ≥ 0.950 is considered indicative of acceptable fit. The Root Mean Square Error of Approximation (RMSEA) was recorded at 0.075, accompanied by a 95% confidence interval ranging from 0.073 to 0.077 (see Table 1).

The Standardized Root Mean Square Residual (SRMR) index, registering a value of 0.070, gauges the distinctions between the implied and empirical variance–covariance matrices. According to Schreiber et al. (2006), a value of 0.08 or below is considered acceptable. Additionally, computed indices encompass a Comparative Fit Index (CFI) of 0.968 and a Tucker-Lewis Index (TLI) of 0.966. Consistent with the criteria outlined by Ruiz et al. (2010) and Schreiber et al. (2006), a CFI and TLI value of 0.95 or higher signifies an adequate fit. Consequently, the model exhibited a satisfactory fit to the sample data, affirming the first-order latent structure comprising six individual factors (see Table 5).

**Table 5:** Fit indices in the compared models.

Model	GFI	RMSEA	SRMR	CFI	TLI
Model I (six factors)	0.977	0.075	0.070	0.968	0.966
Model II (bifactor)	0.957	0.973	0.084	0.079	0.961
Model III (higher-order bifactor)	0.984	0.988	0.051	0.051	0.986

The second analysis (Model II) assessed the fit of the CFA model. The obtained GFI index of 0.973 indicated that 97.3% of the observed variances and covariances were explained by the variance–covariance matrix estimated by the model. The SRMR value of 0.079 gauges the extent of disparity between the implied and empirical variance–covariance matrices, with smaller SRMR values indicating a superior fit. Schreiber et al. (2006) proposed that values below 0.08 are acceptable. Additionally, the CFI value was 0.961, and the TLI value was 0.957; according to Ruiz et al. (2010), an acceptable fit is achieved when CFI and TLI values are equal to or greater than 0.95. Finally, the RMSEA index was 0.084, with a 95% bootstrap confidence interval ranging from 0.082 to 0.086. This suggests a noticeable incongruence between the observed and expected outcomes (refer to Table 1). Therefore, the model comprising a first-order latent structure with six individual factors and a general factor did not adequately fit the sample data because at least one of the fit criteria were not met (see Table 1).

Finally, the fit indices of the higher-order bifactor model (Model III) yield a GFI index of 0.988, indicating that 98.8% of the observed variances and covariances were explained by the variance–covariance matrix estimated by the model. The SRMR value of 0.051 estimates the disparity between the implied and empirical variance–covariance matrices. Additionally, the CFI value was 0.986, and the TLI value was 0.984. Finally, the RMSEA index was 0.051, with a 95% bootstrap confidence interval ranging from 0.049 to 0.053. Thus, the model comprising a latent structure of six individual factors, one general factor, and two higher-order dimensions (Model III) adequately fit the sample data (see Table 6. Values in reference to Table 1).

**Table 6:** Descriptive statistics of the standardized factor loadings in the compared models.

	Model I				Model II				Model III			
Factor	Mean loading	Min loading	Max loading	Range of variation	Mean loading	Min loading	Max loading	Range of variation	Mean loading	Min loading	Max loading	Range of variation
Sensory discomfort (SD)	0.805	0.556	0.947	0.391	0.622	0.486	0.798	0.312	0.586	0.426	0.796	0.370
Aesthetic sensitivity (AS)	0.717	0.626	0.779	0.153	0.367	0.082	0.613	0.531	0.567	0.485	0.698	0.213
Social affective sensitivity (SAS)	0.686	0.510	0.848	0.338	0.441	0.246	0.601	0.355	0.567	0.360	0.694	0.334
Emotional and physiological reactivity (EPR)	0.667	0.510	0.835	0.157	0.528	0.212	0.713	0.501	0.512	0.145	0.726	0.581
Sensory sensitivity to external subtle stimuli (SIES)	0.625	0.532	0.691	0.159	0.295	0.090	0.449	0.359	0.412	0.302	0.542	0.240
Sensory comfort/Pleasure (SC)	0.577	0.395	0.872	0.477	0.522	0.373	0.593	0.220	0.607	0.556	0.652	0.096
General factor (g)					0.481	0.154	0.664	0.510	0.415	−0.022	0.696	0.634

### 3.3 Reliability

A further analysis of Model I focused on examining the reliability of the constituent factors of the S-SPSQ. Test reliability refers to the accuracy with which a test measures a specific psychological trait. The reliability coefficients (McDonald’s $\omega_{3})$ for the S-SPSQ factors ranged from good to excellent ($\omega_{1}$ ∈ [0.752–0.916]), except for the Sensory Comfort/Pleasure (SC) factor, which exhibited a lower value of 0.700 (0.581) (Campo-Arias and Oviedo, 2008). The following provides a breakdown of the reliability of each factor: the Sensory Discomfort (SD) factor is composed of eight items and obtained a reliability coefficient of $\omega_{1}$ = 0.916. The Aesthetic Sensitivity (AS) factor comprised of five items obtained a reliability coefficient of $\omega_{1}$ = 0.799. The Social Affective Sensitivity (SAS) factor, which is composed of eight items, yielded a reliability coefficient of $\omega_{1}$ = 0.844. The Emotional and Physiological Reactivity (EPR) factor, which consists of 11 items, obtained a reliability coefficient of $\omega_{1}$ = 0.874. The Sensory Sensitivity to External Subtle Stimuli (SIES) factor that contains six items yielded a reliability coefficient of $\omega_{1}$ = 0.752. Finally, the Sensory Comfort/Pleasure (SC) factor, which is composed of four items, obtained a reliability coefficient of $\omega_{1}$ = 0.581. The average ordinal reliability coefficient $\omega_{1}$ for the six factors in the questionnaire is 0.794.

The reliability indices obtained for Model III were good. The average inter-factor reliability of the model was 0.787 varying between 0.700 and 0.904 except for the Sensory Sensitivity to External Subtle Stimuli (SIES) factor, which exhibited a lower value of 0.553. Disaggregating by factor we have that: Sensory Discomfort (SD), composed of eight items, obtained a reliability of 0.812; Aesthetic Sensitivity (AS), which contains five items, obtained a reliability of 0.719; Social Affective Sensitivity (SAS), which is composed of eight items, obtained a reliability of 0.794; the Emotional and Physiological Reactivity (EPR) factor, composed of 11 items, obtained a reliability of 0.805; Sensory Sensitivity to Subtle External Stimuli (SIES), composed of six items, obtained a reliability of 0.553, the Sensory Comfort/Pleasure (SC) factor, composed of four items, obtained a reliability of 0.700, and the General Factor, composed of 42 items, obtained a reliability of 0.900. The reliability of the positive dimension (SIES, SC, SAS, and AS) and the negative dimension (EPR and SD), as well as the General Factor (g) of the S-SPSQ were satisfactory $\omega_{3}$ ∈ {0.893, 0.904}, (Table 7).

**Table 7:** Reliability indices in the compared models.

Latent	Cronbach’s *α*	*ω*1 (Omega unidimensional)	Cronbach’s *α*	*ω*2 (Omega hierarchical)	Cronbach’s *α*	*ω*3 (Omega higher-order bifactor)
AS	0.824	0.799	0.824	0.650	0.824	0.719
SAS	0.871	0.844	0.871	0.663	0.871	0.794
SIES	0.791	0.752	0.791	0.469	0.791	0.553
SD	0.929	0.916	0.929	0.837	0.929	0.812
EPR	0.892	0.874	0.892	0.816	0.892	0.805
SC	0.676	0.581	0.676	0.603	0.676	0.700
g	–	–	0.937	0.928	0.937	0.900
POS	–	–	–	–	0.920	0.904
NEG	–	–	–	–	0.926	0.893

The alpha coefficient estimates the reliability of total scores under the conception of essential tau-equivalence tests, assuming a single common construct for all items, equal factor loadings, and uncorrelated measurement errors. Meanwhile, the omega coefficient estimates the reliability of scores for each factor in a bifactorial model or a factorial model under a congeneric approach that does not require equal factor loadings (Zinbarg et al., 2005). The calculation of omega3 uses the sample variance of the observed scores.

### 3.4 Comparison of the models

By incorporating a general factor (g) to the existing six individual factors and two higher order dimensions, the comparative fit index (CFI) increased by 0.018, the SRMR decreased by 0.019, and the RMSEA decreased by 0.024. To determine whether there was a reliable difference in fit between models I and III, the thresholds of |∆ CFI|, |∆ SRMR| and |∆ RMSEA| > 0.01 were chosen as markers of a difference in fit between the two models under study (Chen, 2007). Based on this criterion, it can be concluded that the model with latent structure of six factors, one general factor and two higher order dimensions provided a better fit to the data compared to the six-factor-only model and the six-factor plus one general factor model (Table 8).

**Table 8:** Fit indices in the compared models.

Model	CFI	SRMR	RMSEA (95% CI)
1: 6 factors CFA	0.968	0.070	0.075 (0.073–0.077)
2: bifactor	0.961	0.079	0.084 (0.082–0.086)
3: higher-order bifactor	0.986	0.051	0.051 (0.049–0.053)

## 4 Discussion

The main purpose of this study was to translate, adapt, and validate the Sensory Processing Sensitivity Questionnaire (SPSQ) for Spanish speakers. The research encompassed a series of studies that established a comprehensive model for the S-SPSQ, including six factors and a bi-factor Structure (six factor plus a general a factor). Additionally, a higher-order bifactor model, incorporating positive and negative factors was analyzed. The findings revealed general similarities with De Gucht et al.’s (2022) original measure, including significant positive loadings and consistency in goodness-of-fit indices between the studies.

Expanding on De Gucht et al.’s (2022) research, who used Cronbach’s alpha for internal consistency assessment, the present study employed McDonald’s omega coefficients which offers some advantages, such as providing a more accurate estimation of observed score reliability, accommodating tau-equivalent and congeneric measures, and offering a clearer representation of systematic variance proportion in the scale (Flora, 2020). Furthermore, omega coefficients offer insights into the hierarchical structure of latent factors, enhancing the understanding of multidimensional contexts, aligning with contemporary methodological advancements.

When comparing the three individual models—Model I (Six-Factor Structure), Model II (Bi-factor Structure), and Model III (Higher Order and Bifactor Structure)— the third model demonstrated exceptional fit, with higher order factors indicating improved representation of sensory sensitivity. Thus, Model III emerged as the strongest option for instrument adaptation and validation, in line with De Gucht et al.’s (2022) original findings [CFI = 0.986, SRMR = 0.051, RMSEA (95% CI) = 0.051(0.049–0.053)]. While Model II introduced a general factor, it did not substantially improve fit. In contrast, Model III, with higher order factors, struck a balance between model complexity and improved representation of sensory sensitivity. Most factors demonstrated acceptable levels of reliability, although the SIES factor showed lower reliability (ω = 0.469 in Model II and 0.553 in Model III), suggesting a cautious interpretation. The omega, based on less strict assumptions and adjusted to CFA characteristics, confirmed the alpha reliability results obtained by SPSQ validation.

In sum, Model III demonstrated superior fit and reliability in both studies regarding internal structure. However, one discrepancy was that De Gucht et al. (2022) found the SC (sensory comfort/pleasure) to have the lowest reliability, while we found the SIES (sensitivity to subtle internal and external stimuli) to have the lowest reliability. Future research might further investigate the reliability of factors using both alpha and omega coefficients to get a deeper understanding of both factors to conclude their relevance in the SPSQ structure.

To conclude, the SPSQ and S-SPSQ represent an advancement in SPS measurement, transitioning from unifactorial models of high sensitivity using the HSP scale (Aron and Aron, 1997) to bifactorial models (Evans D. E. and Rothbart, 2007; 2008; 2009; Ershova et al., 2018; Rinn et al., 2018; Lionetti et al., 2019), and additionally herein incorporating a more complex multidimensional model that considers both the positive and negative related aspects of high sensitivity. Despite notable contributions from models and adaptations based on three factors (Smolewska et al., 2006) (AES, LST, and EOE factors), the SPSQ seems appropriate for examining the complexity of SPS. The integration of various positive and negative dimensions allows a more comprehensive conceptualization and assessment of SPS, leading to a deeper understanding of individual differences arising from the SPS trait.

## 5 Limitations

Although the current study’s sample size was smaller than those of previous studies, the results of the current research align with those obtained in the work conducted by De Gucht et al. (2022), demonstrating consistency in the findings and supporting the reliability of the results. Additionally, further analyses on concurrent validity, predictive validity, and work investigating correlations between SPS and personality traits and health outcomes related to SPS, would complement the current investigation. It is also important to conduct studies on item and trait parameter invariance to ensure comparability across different age groups and demographic backgrounds.

Considering these limitations, it is noteworthy that our sample size exhibited sufficient power within the Chilean population to provide significant results. This lends support for the study’s robustness, as evidenced by its adherence to the requisite statistical criteria. The findings of this study, consistent with those reported by De Gucht et al. (2022), provide support for the validity and reliability of the adapted SPSQ as a valuable tool for measuring SPS in Spanish-speaking persons. Future research should address these limitations and explore further applications of the SPSQ in diverse cultural contexts and populations to advance our understanding of SPS and its implications for individuals’ well-being and psychological functioning.

In line with other studies (Smolewska et al., 2006; Ershova et al., 2018; Khosravani et al., 2021; Costa-López et al., 2022; De Gucht et al., 2022), there was a significant gender imbalance in our study, with a considerably higher proportion of female participants (844 female participants representing 83% of the sample). This discrepancy raises potential questions related to gender disparities in psychological studies broadly speaking, and the assessment of SPS as evidenced by previous research (Montoya-Pérez et al., 2019). Studies have consistently shown a higher participation of women compared to men, raising questions about whether the factor structure of high sensitivity applies equally to both genders (Visnes et al., 2022). In the present study, results might be susceptible to influences from prevailing female social stereotypes. The gender imbalance observed in participant representation herein and in other studies of SPS calls for a critical examination of potential gender biases influencing sensitivity research. Thus, future studies might prioritize achieving a more balanced representation of gender to attain a comprehensive understanding of SPS.

Considering the significance of this adaptation and validation process, future research should also aim to extend the application of the SPSQ and explore its psychometric properties in other cultural contexts and populations. Continued investigation will advance the field and foster a more nuanced and accurate comprehension of high sensitivity.

## 6 Conclusion

The outcomes of the present study suggest that the S-SPSQ is a valuable tool for assessing SPS among Spanish-speaking individuals. Unlike traditional approaches that primarily focus on the challenges associated with sensitivity, the SPSQ and S-SPSQ provide a more balanced perspective by considering both positive and negative aspects. This holistic approach enhances the evaluation of SPS’s impact on psychological well-being and informs therapeutic interventions that emphasize the positive facets of sensitivity. This shift in perspective promotes resilience and adaptive coping strategies.

The S-SPSQ contributes to a greater understanding of SPS, suggesting a nuanced perspective of the trait, with one general factor, a six-factor structure, and a factor capturing both positive and negative dimensions of SPS. Thus, this study provides a comprehensive view of individual differences in high sensitivity. It also paves the way for future research and applications in mental health and clinical psychology. For example, the S-SPSQ might be used in clinical practice, allowing clinicians to identify specific sensory dimensions, that are troublesome for the highly sensitive individual, guiding a personalized therapeutic approach. In sum, the S-SPSQ expands on previous work increasing our understanding of SPS and providing a tool that might be utilized to better understand SPS and enhance outcomes for highly sensitive persons.

## Data availability statement

The datasets presented in this study can be found in online repositories. The names of the repository/repositories and accession number(s) can be found at: https://doi.org/10.5281/zenodo.11085238.

## Ethics statement

The research protocol received approval from the Faculty of Health, the Research Institute, and the Ethics Committee of Universidad Central de Chile (Project identification code 120/2022). The studies were conducted in accordance with the local legislation and institutional requirements. The participants provided their written informed consent to participate in this study.

## Author contributions

PS-Q: Conceptualization, Funding acquisition, Investigation, Methodology, Supervision, Writing – original draft, Writing – review & editing. RB-R: Data curation, Formal analysis, Funding acquisition, Methodology, Writing – original draft. BA: Funding acquisition, Supervision, Writing – review & editing. AV-M: Funding acquisition, Methodology, Writing – original draft. MP-C: Funding acquisition, Writing – original draft. AC: Funding acquisition, Writing – original draft.
