# Supplementary material for: Cross-cultural adaptation and validation of the Spanish sensory processing sensitivity questionnaire (S-SPSQ)
Source: Front Psychol. 2024 May 2;15:1279889. doi: 10.3389/fpsyg.2024.1279889 (PMC11098279; doi:10.3389/fpsyg.2024.1279889)
Supplement: Supplementary file 1 [file Presentation_1.pdf]

S-SPSQ (Salinas et al., 2024)
Definitive items (SPANISH)

1. Usualmente siento más frío que otras personas
2. En situaciones estresantes me frustro con facilidad
3. Me siento nervioso cuando muchas cosas suceden de repente
4. A veces logro notar ojos tristes detrás de la sonrisa de una persona
5. Me resulta muy contradictorio cuando el tono de voz de una persona no concuerda con lo que quieren decir sus palabras
6. Las luces muy coloridas, brillantes o intermitentes me generan molestia
7. No me gusta escuchar música a un volumen elevado
8. Los sonidos estridentes o fuertes me parecen muy molestos
9. Los colores muy brillantes me molestan o incomodan
10. Usualmente logro notar el estado o tono emocional de una fotografía u obra de arte
11. Mirar a los ojos de alguien me da una buena idea de si está o no diciendo la verdad
12. Cuando estoy con las personas que quiero me siento extraordinariamente bien
13. Los cambios en mi vida me resultan incómodos
14. Me siento fácilmente molesta/o en medio de multitudes o situaciones caóticas
15. Creo saber cuando las personas fingen no tener miedo
16. Me conmueve con facilidad la música o el arte
17. Siento inmediatamente cuando mi boca o lengua están secos
18. Llaman mi atención detalles visuales imperceptibles para otros
19. Me perturban fácilmente las luces brillantes
20. Disfruto mucho de situaciones divertidas
21. Disfruto con facilidad de los aromas sutiles
22. Realmente puedo disfrutar de una situación relajante
23. Los sonidos muy agudos me parecen molestos
24. Siento que el estado de ánimo de otras personas me afectan con facilidad
25. Rápidamente me doy cuenta de cambios en mi cuerpo, como por ejemplo, cambios en mi temperatura corporal
26. Usualmente noto cuando alguien esconde sus emociones con una sonrisa
27. Usualmente noto olores casi imperceptibles
28. Me estresa hacer muchas cosas en poco tiempo
29. Me molestan las luces demasiado brillantes
30. Frecuentemente soy capaz de sentir hasta las más mínimas contracciones por hambre en mi estómago
31. Usualmente siento los latidos de mi corazón
32. Me molesta el tener que hacer más de una cosa a la vez
33. Soy sensible a la tensión física que experimenta en ocasiones mi cuerpo
34. Me molestan con facilidad los ruidos
35. Entiendo las cosas intuitivamente mejor que otras personas
36. Siento que soy una persona que tiene una rica vida interior
37. Cuando escucho música soy capaz de notar los tonos más sutiles y conmovedores
38. Cuando veo una película inspiradora me envuelve una sensación positiva
39. Me puedo sentir muy conmovido por una bella obra de arte
40. Intento organizar mi vida de tal manera que pueda evitar situaciones molestas o apremiantes
41. Cuando la gente se siente incómoda sé cómo tranquilizarla
42. Me siento incómodo cuando muchas cosas pasan a mi alrededor
43. Cuando me enfrento a una situación competitiva me pongo tan nerviosa/o que lo hago por debajo de mis capacidades
